# Antioxidant, Anti-Inflammatory, and Microbial-Modulating Activities of Nutraceuticals and Functional Foods 2019

**DOI:** 10.1155/2020/6981542

**Published:** 2020-04-02

**Authors:** Ilaria Peluso, Débora Villano Valencia, C-Y. Oliver Chen, Maura Palmery

**Affiliations:** ^1^Research Center for Food and Nutrition, Council for Agricultural Research and Economics (CREA-AN), Rome, Italy; ^2^Universidad Católica San Antonio de Murcia (UCAM), Murcia, Spain; ^3^Biofortis Research, Mérieux NutriSciences, Addison, IL, USA; ^4^Sapienza University of Rome, Rome, Italy

We invited, to this special issue, investigators to contribute research articles based on both original preclinical and clinical data and comprehensive reviews aimed at evaluating the antioxidant and anti-inflammatory activities of nutraceuticals and functional foods, as well as the functional quality assessment of both natural and industrially processed foods. Twenty papers were included, comprising 17 original articles and 3 reviews. The latter discussed the potential health effects of sulforaphane (SFN) (Houghton), barberry (*Berberis vulgaris*) (Kalmarzi et al.), and *Oviductus ranae* (Zhang et al.). In addition to the effects on nuclear factor-erythroid 2-related factor 2 (Nrf2) and nuclear factor-kappa B (NF-*κ*B), SFN has been suggested as a urease inhibitor, potentially reducing the disease risks associated with *H. pylori* infection (Houghton). Immunomodulating activities have been reported for barberry, including the inhibition of inflammatory cytokines (interleukin- (IL-) 1, tumor necrosis factor- (TNF-) *α*, and interferon- (IFN-) *γ*) and the stimulation of IL-4 and IL-10 (Kalmarzi et al.), whereas bioactive components of *Oviductus ranae* increase productions of IL-1*β*, IL-6, and TNF-*α* and activate the NF-*κ*B pathway (Zhang et al.). Therefore, opposite effects on the immune responses can be observed with different bioactives and nutraceuticals.

Among research papers in this special issue, Chen et al. investigated the role of sirtuin 7 (SIRT7), a NAD+-dependent deacetylase, in the NF-*κ*B signaling pathway in an *in vitro* model of lipopolysaccharide- (LPS-) induced inflammation, and NF-*κ*B modulations have been reported for lupeol in streptozotocin-induced hyperglycemic rats (Beserra et al.) and for gallic acid in the ethyl acetate extract fraction of the *Terminalia bellirica* fruit (Chen et al.). Farcas et al. studied both anti-inflammatory and antioxidant activities of *xerophyte Plantago sempervirens Crantz in vitro* and in a turpentine oil-induced inflammation animal model. Antioxidant and antigenotoxic effects were reported for methanol extracts of the edible mushroom northwestern Turkey Infundibulicybe geotropa (Bull.) Harmaja (Sevindik et al.). Górnicka et al. found that *α*-tocopherol reduced lipid peroxidation in rats subjected to a training protocol on a treadmill. On the other hand, Nrf2-mediated antioxidant effects have been reported for a heteropolysaccharide extracted from the brown alga *Sargassum fusiforme* in a *Drosophila melanogaster* model (Zhang et al.), and a heat-resistant extract from the edible microalga Aphanizomenon flos-aquae (AFA) has been proposed by Nuzzo et al. as a functional food for protection against oxidative stress (Xiao et al.). Nrf2 was also involved in the inhibition of oxidative stress by indigo naturalis. The latter also reduced Th1/Th17 responses in a DSS-induced colitis model in mice (Xiao et al.). A polyphenolic extract of *Chrysanthellum americanum* has been studied by Cojocariu et al. in a rat model of irritable bowel syndrome. Lv et al. suggested a systems pharmacology and microbiota approach for treatment of inflammatory bowel disease, including an absorption-distribution-metabolism-excretion (ADME) analysis of potential active compounds. Azzini et al. studied the differences in phytochemical profiles and antioxidant activities of different S. Giovanni varieties (Pyrus communis L.). Palmacci et al. reported that the inflammatory marker neutrophil-to-lymphocyte ratio (NLR) was lower in celiac disease patients who consumed chocolate. Green tea (GT) and GT extract reduced inflammation in a high-fat diet mouse model (Torres et al.) and ischemia-induced retinal ganglion cell degeneration in rats (Yang et al.), respectively. Antioxidant and anti-inflammatory activities were reported for thymoquinone (TQ) and/or piperine (PP) against microcystin-LR-induced hepatotoxicity and neurotoxicity in mice by Abdel-Daim et al., whereas Kim et al. tested the ethyl acetate extract of Salicornia europaea L. and its bioactive compound irilin B in the 1-methyl-4-phenyl1,2,3,6-tetrahydropyridine- (MPTP-) intoxicated Parkinson's disease- (PD-) like mouse model.

In conclusion, this issue includes original and review articles covering many aspects of the antioxidant and anti-inflammatory activities of nutraceuticals, functional foods, and bioactive compounds from various origins.

*Ilaria Peluso* *Débora Villano Valencia* *C-Y. Oliver Chen* *Maura Palmery*

